# Determine the BMI levels, self-concept and healthy life behaviours of children during a school based obesity training programme

**DOI:** 10.3934/publichealth.2020043

**Published:** 2020-07-15

**Authors:** Naime Altay, Ebru Kılıcarslan Toruner, Ebru Akgun-CITAK

**Affiliations:** Gazi University, Health Sciences Faculty Nursing Department, Ankara, Turkey

**Keywords:** obesity training program, self-concept, healthy life behavior, children

## Abstract

Sedentary lifestyles and unhealthy nutrition, in particular, cause childhood obesity. The purpose of this semi-experimental research is to determine the changes in body mass index, self-concept, and healthy lifestyle behaviours of children during a training programme to prevent obesity. Children 9 and 15 years old were included from two public secondary schools. A total of 1609 students completed the study in the intervention and control groups (1022 vs 587, respectively). The training programme for the intervention group at the schools had three sessions in 12 weeks and was about obesity, body mass index (BMI) calculation, a healthy lifestyle and coping with stress. Data was collected through a sociodemographic data form, healthy lifestyle behaviours data form, Piers-Harris Children's Self-Concept Scale and weight-height measurements during the first and last weeks of the programme (except for the sociodemographic form). Before training, BMI's of children in the intervention group were higher than in the control group (19.61 ± 3.8 vs 19.00 ± 3.5, respectively). The gap between BMI scores of the groups was narrowed after the training (p > 0.05). The mean score on the Self-Concept Scale increased in the intervention group after the training (63.21 ± 9.5) as compared to before the training (61.16 ± 10.4); whereas in the control group, there were no differences found (p = 0.908). In the intervention group, the number of children who had breakfast after the training (81.1%) increased compared to before the training (74.1%) (p = 0.001). The trainings were provided to students to increase healthy nutrition, physical activity and to decrease sedentary lifestyles.

## 1. Introduction

Education programmes related to healthy life behaviours have an important place to reduce preventable diseases and deaths. Sedentary lifestyles and unhealthy nutrition cause the death of millions of people all around the world. Sedentary lifestyles are gradually increasing among children. Research shows that the time that school-age children spend in front of the television or computer ranges between 7.4–9.8 hours/day [1]–[4].

In primary school students, inadequate nutrition and malnutrition problems, such as not having the habit of eating breakfast every day [5], consuming fast food at least once a day and skipping meals are frequently observed [1],[3],[6]. The ratio of physical activity and healthy nutrition [7] for children who spend most of their time in front of a television and/or computer is also low [8]. It is found that as the daily time children spend in front of a computer increases, their BMI (Body Mass Index) also increases; however, as the daily time they spend on physical activity increases, the value of BMI decreases [8],[9].

In particular, sedentary lifestyles and unhealthy nutrition cause childhood obesity. Obesity in children is a notable health problem in many countries in the world today [10],[11]. The World Health Organization (WHO) Europe Area Office denotes that the rate of being overweight for children and adolescents in the European area is around 20% and one in two of these individuals is obese [11]. Being overweight can cause various physical and psychological problems in children [12], such as Type-2 diabetes, cardiovascular system problems, hypertension and respiratory system problems (such as sleep apnea), as well as some cancer and osteoarthritis. Low self-esteem and body image, eating disorders and a decrease in the quality of life are wide-spread psychological problems [8]. There is also a negative impact from higher BMI levels on the perception of self-concept in children [13],[14]. Self-concept is related to the perception of how people view themselves in physical, psychological and social aspects [15]. It is a dynamic process and can therefore be expanded by perception and understanding of personal and social values. In other words, self-concept in children could be increased by providing opportunities, such as education and physical activity [16],[17]. (Positive self-concept is promoting the achievement of desirable outcomes like competence, social skills, coping with stress, long-term health and well-being [18]–[21]). If we could improve the self-concept of children with obesity prevention programmes, we could promote healthy lifestyles for children. As a result of the escalation of habits that negatively impact health and co-morbidities in children, it has been demonstrated that health professionals need to focus on the prevention of this problem.

Apart from education, schools have an important role in developing children's health and in the prevention and reduction of risky health behaviours [22]. Through healthy lifestyle programmes conducted in schools, healthy nutrition and physical activity behaviours in children can be improved and sedentary lifestyles can be minimised. There are various school-based programmes in existence [23]–[25]. There is limited published intervention research on children in schools that targets improvement of healthy lifestyle behaviours, BMIs, and psychosocial aspects. Also, most studies have searched indicators of psychological aspects of overweight and obesity, such as anxiety, depression and self-esteem after training programmes. However, little is known about alterations in self-concept during the programmes. Therefore, one of the aims of this study is to determine whether the training programme has a positive impact on children's perceptions of self-concept and body mass index. There is a need for programmes to improve healthy lifestyle habits in children and to evaluate the efficiency of the existing programmes. Through healthy lifestyle programmes, physical and mental development of school-age children can be accomplished in the best possible way.

### 1.1. Purpose

This research is conducted to determine the changes in body mass index, self-concept, and healthy lifestyle behaviours of children during a school based training programme to prevent obesity.

### 1.2. Research questions

For children in intervention and control groups before and after the applied training programme:

1. Is there a difference between their body mass indexes?

2. Is there a difference between the averages of their self-concept scale points?

3. Is there a difference between their healthy lifestyle behaviours?

4. Is there a correlation between BMI and the average of self-concept scale points?

## 2. Methods

### 2.1. Design and participants

This is a semi-experimental research conducted to secondary school students (5^th^, 6^th^ and 7^th^ graders) in order to prevent obesity.

**Figure 1.**
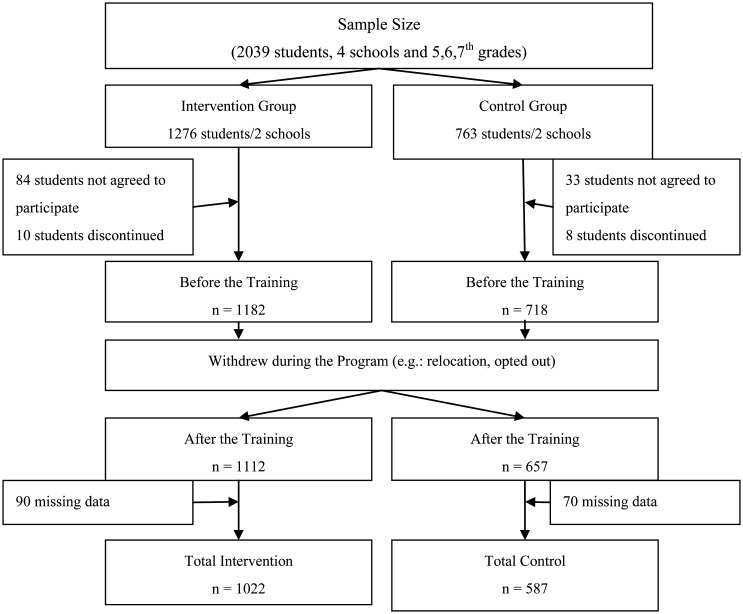
The sample size of the research.

It is not possible to differentiate secondary schools within the boundaries of the Ankara Metropolitan Municipality based on their socioeconomic levels according to the State Statistics Institute. Therefore, by an improbable sampling method, two regions in the Ankara Province and two public schools from each region were selected. As the research is semi-experimental, one school from each region was used as the intervention group and the other as the control group according to student numbers. We preferred a greater population for the intervention group to determine the effect of trainings. The criteria for children to be included in the research were: a) registered in 5^th^, 6^th^ or 7^th^ grade; b) personal and parental consent to participate in the activity; and c) able to continue in the training programme. The criteria for exclusion from the research were chronic disease and obesity due to physiological reasons. There were 2039 students who fit the research criteria and were included in the research; the research was completed with 1609 students. There were no students excluded because the students with chronic illness or obesity problems did not agree to participate in the study in the first place. The sample size of the research is given in Figure 1.

### 2.2. Data collection forms/tools

The research data was collected through the use of three forms.

The Sociodemographic Data Form consisted of five questions that were prepared by researchers to identify sociodemographic characteristics of children and families (age, gender, number of children in family, employment status of mother, economic status of family). The Healthy Lifestyle Behaviours Data Form consisted of eight close-ended questions (having breakfast, eating in front of screen, time spent in front of screen, physical activity time per day) about behaviours that are considered to be effective on obesity in children. At the end of each question, the “other” option is provided. The forms were developed by researchers [23]–[26].

The Piers-Harris Children's Self-Concept Scale was developed by Piers-Harris (1964) and adapted to Turkish by Öner (1996) and Çataklı (1985). It aims to evaluate children's opinions, emotions and attitudes towards themselves. The scale that is answered by either “Yes” or “No” consists of 80 items with subscales of “Behaviour”, “Intellectual/School status”, “Physical appearance”, “Anxiety”, “Popularity” and “Happiness”. Answers are graded with a key and points range between 0 and 80. Higher points indicate positive self-conception and lower points indicate negative self-conception. The reliability coefficient of the scale for the subscales range between 0.78 and 0.93. The reliability coefficient of the Turkish form ranges between 0.81 and 0.89 [27].

### 2.3. Data collection

The programme was applied for a total of 12 weeks during spring semester. During the first week of the programme, the sociodemographic data form, healthy lifestyle data form and Piers-Harris Children's Self-Concept Scale were completed in the intervention and control groups, and weight and height measurements were taken. In the 2^nd^, 4^th^ and 6^th^ weeks of the programme, students in the intervention group were given three training sessions. In the 12^th^ week of the programme, all forms except the sociodemographic data form were again completed in both groups and weight and height measurements were taken.

Weight and Height Survey: Weight and height scanning for students was completed by the researchers with the help of nursing students (n:29) who had taken courses on paediatric nursing. Nursing students were provided with an applied demonstration using measurement checklists for almost an hour before the activity. Weight measurement: For measuring the weight of children, a ± 100 g electronic precision scale was developed. Shoes and coats/jackets of students were taken off during the measurement. Height measurement: Portable stadiometer was used. Measurement was completed after taking off student shoes and students were positioned to stand with their backs against the wall, with their feet together, and as upright as possible. The value found for weight and height measurements of children was rounded up to the closest 0.1 cm value. Weight and height measurement values were recorded in the findings records form and BMIs of students were calculated.

### 2.4. Application of the training programme

On the first day of the training, survey forms were administered to intervention and control groups in their classes. The intervention group was provided with three training sessions once every two weeks by all researchers. The length of intervention for each group is a total of 6 weeks. Each training session averaged 40 minutes. Trainings were provided in the conference room of each school to be separate for each class. The size of the training groups ranged between 25–30 students. Each training was provided to 40–45 groups and a total of 1112 students participated in the trainings.

The training content of the research is given in Table 1. Face-to-face and interactive classroom teaching methods with discussions were used during the trainings. Three booklets were prepared by researchers for the trainings. Students in the control group were provided with training booklets only after all of the research data was collected.

**Table 1.** Trainings and Content.

Names and Aims of the Training	Content	Training Methods
I. Training: Am I at the Healthy Weight? Aim of First Training: To teach students the reasons and effects of being overweight and to calculate body mass index	- Causes of being overweight - Factors that affect being overweight - Effects of being overweight - Calculation of body mass index and use of BMI chart	- Discussing the causes of being overweight and their effects with students - Calculating BMI interactively on the training brochure and having children evaluate their own weight
II. Training: 5 Ways of Healthy Life for Children Aim of Second Training: To select two behaviours to change eating habits to prevent obesity and to set up weekly individual exercise programmes. Supporting increased self-esteem by enabling students to make their own programmes.	- Healthy eating habits to prevent obesity - Organising daily activities - Benefits of exercising and its duration - Effects of sedentary life on health	- At least 2 behavioural determinations in which students are expected to change eating habits for preventing obesity They must write their goals in the training brochure - Each student create an individual weekly exercise programme and writes it in the training booklet
III. Training: Effects of Stress on Obesity in Children and Coping with Stress. Aim of Third Training: To teach students how to deal with stress. Also students' self-knowledge will be supported to increase self-esteem.	- Stress - Effects of stress on health - Reducing emotional eating behaviour - Healthy coping methods	- Students should identify the causes of stress in themselves and write them in the training booklet - Discussing coping methods

### 2.5. Data analysis

The data obtained in this research was evaluated with the SPSS 16.0 package programme. Frequency and percentage distribution regarding the data are given. The correlation between variables was measured at the categorical level and analysed with the Chi-Square test. In the case of two groups, the Mann-Whitney U test was used for intergroup comparisons. For pre–post comparisons in dependent groups, the Wilcoxon signed rank test was used. Correlation coefficients and statistical significance for the relationship between non-normally distributed variables was calculated with the Spearman test. Correlation coefficients were evaluated with 0.05–0.30 as low or insignificant correlation, 0.30–0.40 as low to mid-level correlation, 0.40–0.60 as mid-level correlation, 0.60–0.70 as good level correlation, 0.70–0.75 as very good level correlation and 0.75–1.00 as perfect correlation. The level of significance is 0.05 [28].

### 2.6. Ethics consideration

The verbal consent from participating students and written consent from their parents were received. Also written consent from the Ministry of National Education (approved by programme and ethical department of the Ministry) was received for schools.

## 3. Results

Descriptive data regarding the child and family is given in Table 2. The sociodemographic variables found were similar. In the intervention group, the number of children who had breakfast after the training (81.1%) increased compared to before the training (74.1%) (p = 0.001), whereas in the control group, the number of children who had breakfast after training (91.3%) decreased compared to before the training (92.7%) (p = 0.001). Eating in front of a screen decreased significantly after training in both groups compared to before (p = 0.001) (Table 3). When children's behaviours of physical activity and sedentary life are considered, it is found that the number of children who have an hour or more of daily physical activity in the intervention and control groups after the training (intervention 72.8%, control 71.2%) increased significantly (p < 0.05) compared to before the training (intervention 62.8%, control 62.9%). The number of children spending 2 hours or less in front of a screen in the intervention and control groups after training (intervention 58.5%, control 69.0%) significantly increased (p < 0.05) compared to before the training (intervention 1.0%, control 67.0%) (Table 3).

**Table 2.** Descriptive characteristics of children and family.

	Intervention		Control	
Characteristic*	n	%	n	%
Child age (X ± SD) (Min-Max)	11.9 ± 1.01	9–15	11.7 ± 0.82	9.5–15
Child Gender				
Female	508	49.7	294	50.5
Male	514	50.3	293	49.9
Child number in family				
1 child	69	6.8	37	6.3
2–3 children	421	41.2	445	75.8
4 and more children	532	52.0	105	17.9
Mother's working condition				
Working	103	10.0	61	10.3
Not working	919	90.0	526	89.7
Family income				
Expenses is more	34	3.3	26	4.4
Income is equal to expenses	648	63.4	357	60.8
Income is more	340	33.3	204	34.8

* Children's own expressions.

**Table 3.** Comparison of children's healthy life behaviors.

	Before Training					After Training				
	Intervention		Control			Intervention		Control		
Characteristic*	n	%	N	%	Between Groups p	n	%	n	%	Between Groups p
Having breakfast										
Yes	757	74.1	544	92.7	X^2^ = 70.226	829	81.1	536	91.3	X^2^ = 39.803
No	265	25.9	43	7.3	P = 0.001	193	18.9	51	8.7	p = 0.001
	In-groups p Intervention: X^2^ =144.680; p = 0.001					In-group p Control: X^2^ = 73.69; p = 0.001				
Eating in front of screen										
Yes	564	55.2	380	64.7	X^2^ = 6.760	559	54.7	363	61.8	X^2^ = 15.463
No	458	44.8	207	35.3	p = 0.009	463	45.3	224	38.2	p = 0.001
	In-group p Intervention: X^2^ = 148.71; p = 0.001					In-group p Control: X^2^ = 41.0; p = 0.001				
Physical Activity Time per Day										
Less than 1 hour	380	37.2	218	37.1	X^2^ = 0.001	278	27.2	169	28.8	X^2^ = 0.469
1 hour and more	642	62.8	369	62.9	p = 0.986	744	72.8	418	71.2	p = 0.493
	In-group p Intervention: X^2^ = 147.973; p = 0.001					In-group p Control: X^2^ = 49.353; p = 0.001				
In front of screen per Day										
Less than 2 hour	521	51.0	393	67.0	X^2^ = 49.53	598	58.5	405	69.0	X^2^ = 11.221
2 hour and more	501	49.0	194	33.0	p = 0.001	424	41.5	182	31.0	p = 0.001
	In-group p Intervention: X^2^ = 58.504; p = 0.001					In-group p Control: X^2^ = 57.15; p = 0.001				

* Children's own expressions.

The BMI of children in the intervention group before the training (19.61 ± 3.8) is found to be significantly more (p = 0.003) compared to children in the control group (19.00 ± 3.5). However, there is no significant difference identified between BMI of children in the intervention and control groups after the training (p > 0.05) (Table 4). The average Piers-Harris Self-Concept Scale point in the intervention group after training (63.21 ± 9.5) increased significantly (p = 0.001) compared to before training (61.16 ± 10.4), whereas in the control group, no differences were found (p = 0.908) (Table 4).

## 4. Discussion

In our research, in the intervention group, the number of students who had breakfast after training increased compared to before the training, whereas in the control group, the number of students who had breakfast after the training decreased compared to before the training. Moreover, eating in front of a screen has significantly decreased after training for both groups. In other similar studies, it has been found that training programmes provided to students have an impact on the increase in their eating habits, level of knowledge on food groups and appropriate food management for lunch [10],[29]–[31]. It has been demonstrated that compared to before the training, there is improvement in children's knowledge on nutrition and feeding habits after the health care programme that was applied [31].

In our research, the number of children engaging in an hour or more of daily physical activity increased significantly after training in both groups compared to the number of children spending 2 hours or less in front of the screen in the intervention and control groups after training. Amaya-Castellanos et al. (2015) found that children's information on physical activity has significantly increased [10]. In our study, it is thought that it would be effective for students to calculate their body mass indexes and to make individual decisions regarding nutrition and exercise.

In our study, the BMI levels of children in the intervention group did not show a reduction after the programme. Sbruzzi et al. (2013) found in their systematic review with meta-analysis of a randomised clinical trial that educational interventions were significantly effective to reduce BMI in treatment but not for prevention of obesity in children 6–12 years old [32]. Reinehr et al. (2010) investigated the long-term outcomes of lifestyle interventions over 5 years in children 4–16 years old. They found that the reduction in BMIs of obese children was greater after 4 years of the intervention than after one year [33]. On the other hand, another systematic review showed that there was strong evidence to support beneficial effects of child obesity prevention programmes on BMI in children 6–12 years old [34].

Self-concept is a multidimensional construct influenced by a variety of factors. In our study, children's self-concept was increased after the programme in the intervention group. However, the self-concept of children in the control group did not change after the programme. Similar to our finding, Velez et al. (2010) found that self-concept of healthy and overweight adolescents increased after 12-weeks of a resistance training programme [26]. Weight-control interventions generally related to improved self-concept in children and adolescents at the end of the treatment [35]. Taken together, our findings suggest that involvement in a healthy lifestyle programme that includes building goals, learning healthy lifestyles, evaluating their own BMI and learning coping skills may contribute to documented improvements in self-concept among children.

**Table 4.** Comparison of results of the before and after the training BMI and piers-harris self concept scale of children in the intervention and control group.

Groups	Before Training			After Training				p
	BMI			BMİ				
	M ± SD	Min-Max.	Median	M ± SD	Min-Max.	Median	Wilcoxon Z	
Intervention (n = 1022)	19.61 ± 3.8	11.2–38.2	18.87	20.01 ± 4.0	9.8–40.0	19.29	−9.58	0.001
Control(n = 586)	19.00 ± 3.5	8.5-33.1	18.34	19.62 ± 3.7	9.9–36.0	18.84	−10.19	0.001
	*M-U = 272765.5; p = 0.003			*M-U = 283649.5; p = 0.088				

Groups	Self-Concept Scale			Self-Concept Scale				p
	M ± SD	Min-Max.	Median	M ± SD	Min-Max.	Median	Wilcoxon Z	
Intervention (n = 1022)	61.16 ± 10.4	21–77	64.0	63.21 ± 9.5	19–80	65.0	−7.87	0.001
Control(n = 586)	61.94 ± 10.0	17–80	65.0	62.18 ± 10.2	13–78	65.0	−0.116	0.908
	*M-U = 282909.5; p = 0.057			*M-U = 285357.5; p = 0.103				

*M-U: Mann-Whitney U test.

There is a risk for the development of poor self-esteem and concept in overweight children. A group of factors could affect poor self-esteem in children, including cultural thoughts about ideal body shapes, peer victimisation, low or greater parental control and social anxiety [35]. Although there was no statistically significant relationship between children's BMI levels and self-concept in our study, it was determined that self-concept decreased as the BMI levels of children in both groups increased. Poulsen et al. (2011) searched the BMI levels and self-concept of children who were overweight and non-overweight and stated that overweight children had lower scores on self-concept than non-overweight children [36].

The limitation of this study is that self-concept could change from one individual to another. On the other hand, healthy lifestyle data was collected through students' self-expression. Therefore, answers could be wisely chosen and the results could not be generalised. Also, this study was conducted in the spring term of the education. This could result in increased time spent on physical activity. In this study, we evaluated the changes of BMI levels of students during the programme. These changes were not calculated on individual bases, BMI z-scores, and potential influences as age, gender, socioeconomic status.

## 5. Conclusion

In our research, we find that there is an increase in the ratio of children who have breakfast and engage in physical activity at least one hour or more every day in the intervention group after the training programme. The average Piers-Harris Self Concept Scale scores and student habits of eating in front of the screen showed a decrease.
